# Combined pars plana glaucoma drainage device placement and vitrectomy using a vitrectomy sclerotomy site for tube placement: a case series

**DOI:** 10.1186/s12886-021-01872-z

**Published:** 2021-02-25

**Authors:** Enchi Kristina Chang, Sanchay Gupta, Marika Chachanidze, John B. Miller, Ta Chen Chang, David A. Solá-Del Valle

**Affiliations:** 1 Massachusetts Eye and Ear, Department of Ophthalmology, Harvard Medical School, Boston, MA USA; 2 Massachusetts Eye and Ear, Department of Ophthalmology, Boston, MA USA; 3 Bascom Palmer Eye Institute, Miami, FL USA

**Keywords:** Glaucoma, Pars plana, Pars plana glaucoma drainage device, Pars plana vitrectomy, Ahmed drainage implant, Baerveldt drainage implant, Safety, Efficacy

## Abstract

**Purpose:**

The purpose of this study is to report the safety and efficacy of pars plana glaucoma drainage devices with pars plana vitrectomy using one of the vitrectomy sclerotomy sites for tube placement in patients with refractory glaucoma.

**Methods:**

Retrospective case series of 28 eyes of 28 patients who underwent combined pars plana glaucoma drainage device and pars plana vitrectomy between November 2016 and September 2019 at Massachusetts Eye and Ear. Main outcome measures were intraocular pressure (IOP), glaucoma medication burden, best corrected visual acuity, and complications. Statistical tests were performed with R and included Kaplan-Meier analyses, Wilcoxon paired signed-rank tests, and Fisher tests.

**Results:**

Mean IOP decreased from 22.8 mmHg to 11.8 mmHg at 1.5 years (*p* = 0.002), and mean medication burden decreased from 4.3 to 2.1 at 1.5 years (*p* = 0.004). Both IOP and medication burden were significantly lower at all follow-up time points. The probability of achieving 5 < IOP ≤ 18 mmHg with at least 20% IOP reduction from preoperative levels was 86.4% at 1 year and 59.8% at 1.5 years. At their last visit, three eyes (10.7%) achieved complete success with IOP reduction as above without medications, and 14 eyes (50.0%) achieved qualified success with medications. Hypotony was observed in 1 eye (3.6%) prior to 3 months postoperatively and 0 eyes after 3 months. Visual acuity was unchanged or improved in 23 eyes (82.1%) at their last follow-up. Two patients had a visual acuity decrease of > 2 lines. Two eyes required subsequent pars plana vitrectomies for tube obstruction, and one eye had transient hypotony.

**Conclusions:**

The results of pars plana glaucoma drainage device and pars plana vitrectomy using one of the vitrectomy sclerotomy sites for tube placement are promising, resulting in significant IOP and medication-burden reductions through postoperative year 1.5 without additional risk of postoperative complications. Inserting glaucoma drainage devices into an existing vitrectomy sclerotomy site may potentially save surgical time by obviating the need to create another sclerotomy for tube placement and suture one of the vitrectomy ports.

## Background

Glaucoma drainage devices (GDDs) are increasingly used in the treatment of glaucoma refractory to medical therapy or after unsuccessful trabeculectomy or laser procedures. Between 1995 and 2004, the use of GDDs increased by 184% in Medicare patients alongside a 53% decrease in the number of trabeculectomies in eyes without scarring [1]. GDDs may be inserted into the anterior chamber, sulcus, or pars plana depending on comorbid ocular pathology that may preclude placement in a particular location or if a vitrectomy is needed for retinal pathology. Namely, tube placement in the anterior chamber may not be recommended in the setting of some corneal diseases, iridocorneal angle abnormalities, or peripheral anterior synechiae, amongst other pathologies [2]. In these cases, tube placement in the pars plana (PP) may be considered to prevent postoperative anterior chamber complications [3]. PP GDD insertion requires concurrent or prior pars plana vitrectomy (PPV) to prevent vitreous occlusion of the tube. In the combined PPV and PP GDD surgery, treatment of concurrent posterior segment diseases may also be performed, minimizing the need for multiple separate surgical procedures.

During combined PPV and pars plana GDD placement, the GDD tube may be inserted into a new sclerotomy or an existing sclerotomy used for one of the vitrectomy ports [4, 5]. With prior 20-gauge sclerotomies for PPV, concerns regarding the risk of hypotony from leakage around the tube led to greater adoption of creating a new sclerotomy for the tube. In fact, tube insertion into sclerotomies larger than 21-gauge was suggested to be associated with higher leakage rates [6]. Thus, the vitrectomy sclerotomies were closed with suturing to allow for a watertight closure, and a new, separate sclerotomy was created for the GDD tube.

With modern small-gauge (i.e. 23- and 25-gauge) PPVs, however, there may be a lower risk of hypotony from leakage around a tube inserted into a preexisting sclerotomy [3, 7]. Utilizing an existing sclerotomy also likely saves surgical time by eliminating the need to create another sclerotomy and suture one of the port sites, which may result in less inflammation by minimizing the amount of suture material in the sclera. To the best of our knowledge, prior studies have not examined the safety and efficacy of inserting GDDs into an existing sclerotomy utilized by the small-gauge PPVs, thus eliminating the need for creating a new sclerotomy for the tube. In this study, we evaluate the surgical outcomes and complication rates of pars plana GDD placement with tube insertion into one of the existing 23- or 25-gauge vitrectomy sclerotomy sites in patients with refractory glaucoma.

## Methods

### Study design

This is a retrospective cohort study of consecutive adult glaucoma patients who underwent PP GDD insertion with PPV using one of the vitrectomy sclerotomy sites for tube insertion. After receiving approval from the Mass General Brigham Institutional Review Board, medical records of patients who underwent the procedure between April 2016 and November 2019 at Massachusetts Eye and Ear were identified and reviewed. GDD insertion was performed by 9 different providers, and PPV was performed by 8 different providers. Data collection abided by the Declaration of Helsinki and the Health Portability and Accountability Act. Patients were included if they met the following criteria: (1) diagnosis of glaucoma; (2) concurrent PP GDD surgery and PPV with tube insertion into one of the vitrectomy sclerotomy sites; (3) at least 3 months of follow-up; and (4) age ≥ 18 years. If patients had undergone procedures in both eyes, the left eye was included in our study.

Demographic and preoperative data included patient age, gender, glaucoma diagnosis and stage, previous ocular surgeries, IOP, number of glaucoma medications, and visual acuity (VA). Glaucoma stages were defined as circumpapillary retinal nerve fiber layer thinning on optical coherence tomography with Humphrey visual field findings of no abnormalities for mild glaucoma; a single corresponding inferior or superior deficit for moderate glaucoma; or a combination of paracentral or superior and inferior defects for severe glaucoma [8]. An indeterminate stage was defined if visual field testing could not be performed reliably or if the patient had light perception or no light perception vision. IOP was measured with Goldmann applanation tonometry. Preoperative IOP, medication burden, and VA were calculated as an average of the values from two consecutive visits prior to the procedure. Postoperative data were collected at 1 day (POD1), 2 weeks (POW2), 6 weeks (POW6), 3 months (POM3), 6 months (POM6), 1 year (POY1), and 1.5 years (POY1.5) after surgery. At each time point, the IOP, number of glaucoma medications, VA, duration of follow-up, subsequent IOP-lowering procedures, and the presence of postoperative complications such as inflammation in the anterior chamber, hypotony, corneal edema, cystoid macular edema (CME), vitreous hemorrhage, and tube obstruction were recorded. Glaucoma medication burden was obtained at each visit by manually counting the number of individual glaucoma medications used by the patient at that time, with medications composed of two IOP-lowering compounds recorded as two medications.

### Surgical procedure

The procedures were performed by multiple glaucoma and retina specialists. All patients underwent either Ahmed (New World Medical, Rancho Cucamonga, CA, USA) or Baerveldt (Johnson & Johnson Vision, Santa Ana, CA, USA) GDD implant placement with 23- or 25-gauge vitrectomy. The type of glaucoma implant and vitrectomy gauge were at the surgeons’ discretion. For patients with preexisting vitreous hemorrhage or neovascular glaucoma, intravitreal injections were administered preoperatively at the retina surgeon’s discretion. A retrobulbar block was placed by anesthesia, and the operative eye was prepared in the standard ophthalmic fashion. A conjunctival peritomy was created, and sub-Tenon’s space was accessed. The GDD implant was placed and secured to the sclera with 2 interrupted 8–0 nylon sutures. If an Ahmed glaucoma drainage device was used, the tube was first primed with balanced salt solution to open and wet the valve leaflets. If a Baerveldt glaucoma drainage device was used, an external ligation with absorbable suture around the tube was applied. For the vitrectomy, trocars were used to place cannulas in the inferotemporal, superotemporal, and superonasal quadrants through the pars plana in a beveled fashion. A 4 mm infusion cannula was placed through the inferotemporal cannula, and a complete standard three-port PPV was performed. The inferotemporal and superonasal sclerotomies were sutured using 7–0 polyglactin sutures in an interrupted fashion, and the conjunctiva was closed in conjunction with the sclerotomy closure. The superotemporal sclerotomy was then used for GDD tube placement. The tube was then cut at the appropriate length, bevel down. The superotemporal trocar was then removed. If an Ahmed glaucoma drainage device was placed, approximately 0.3 mL of a dispersive ophthalmic viscosurgical device was injected into the pars plana or anterior chamber to prevent hypotony. The tube was then inserted into the pars plana. A cadaveric corneal patch graft was used to cover the tube and secured to the sclera with 2 interrupted 7–0 polyglactin sutures. The overlying Tenon’s and conjunctiva were secured at the limbus using interrupted and running 8–0 polyglactin sutures. A sample video of the surgical procedure is included in the references [9].

### Outcome measures

Primary outcome measures were IOP reduction, glaucoma medication burden, VA, cumulative success probabilities from Kaplan-Meier (KM) analyses, complication rates, and need for additional glaucoma surgery. Success criteria were obtained from the Tube Versus Trabeculectomy Study [10], with the addition of a more-stringent IOP criteria as follows. Success was defined as an IOP reduction ≥ 20% from preoperative IOP without hypotony and (Criteria 1) IOP ≤ 21 mmHg; (Criteria 2) IOP ≤ 18 mmHg; or (Criteria 3) IOP ≤ 14 mmHg. Hypotony was defined as IOP ≤ 5 mmHg. A failure was recorded if a patient did not meet the specified success criteria on two consecutive follow-up visits after 3 months, required additional glaucoma surgery or laser procedure, or developed no light perception (NLP) vision. Patients who required an additional non-glaucoma procedure were censored from survival analysis.

Patients were recorded as a complete success if they satisfied Criteria 1 without medications; qualified success if they satisfied Criteria 1 with medications; and qualified failure if they did not meet the above criteria for success but did not require additional glaucoma surgery or develop NLP vision at their last visit.

### Statistical analysis

Statistical analyses were performed using R (version 4.0.2). A *p* value < 0.05 was considered statistically significant. Average and standard deviation (SD) were calculated for IOP, medication burden, and VA. Line graphs of average values were generated with error bars representing standard deviation. Comparisons with preoperative values were conducted with Wilcoxon paired signed-rank tests. Kaplan-Meier analyses were used to generate cumulative success probabilities, with success criteria as defined above. Hazard ratios for preoperative and demographic characteristics were obtained from Cox proportional hazard regression analyses. Snellen visual acuities were converted to logarithm of minimum angle of resolution (LogMAR) equivalents, with values of 2 and 3 representing count fingers and hand motion vision, respectively. Patients with light perception or no light perception vision were not converted to logMAR equivalents and excluded from mean calculations and paired Wilcoxon testing.

## Results

A total of 28 eyes of 28 glaucoma patients were included in this study. Patient demographic and preoperative data are presented in Table 1. Mean ± SD age was 61.9 ± 20.2 (range 18–90), and 53.6% of the patients were female. Most patients (53.6%) had severe glaucoma, and the most common glaucoma type was chronic angle-closure glaucoma (32.1%) followed by mixed mechanism (25.0%) and primary open-angle glaucoma (21.4%). The mean preoperative IOP was 22.8 ± 7.3 mmHg (range 10.5–38 mmHg) with a medication burden of 4.3 ± 1.0 (range 2–6). The mean follow-up time was 17.7 ± 7.7 (range 5.9–35.5 months). The most common surgical indications for the combined procedure were chronic angle-closure glaucoma with synechiae (32.1%), aphakia with vitreous in the anterior chamber (21.4%), and an anterior chamber intraocular lens (17.9%). For the aphakic eye with vitreous in the anterior chamber, a secondary intraocular lens was not implanted during surgery. The GDD was inserted into an existing sclerotomy site used for the vitrectomy in all eyes.

**Table 1:** Demographic characteristics and preoperative data of glaucoma patients who underwent pars plana glaucoma drainage device implantation with pars plana vitrectomy using the vitrectomy sclerotomy for tube insertion

Parameters	
*Demographics*	
Eyes	28
Female Eyes, N (%)	15 (53.6)
Age (years)	
Mean ± SD	61.9 ± 20.2
Range	18–90
*Glaucoma Stage, N (%)*	
Mild	4 (14.3)
Moderate	7 (25.0)
Severe	15 (53.6)
Indeterminate	2 (7.1)
*Glaucoma Type, N (%)*	
Aphakic	1 (3.6)
Chronic angle closure	9 (32.1)
Mixed mechanism^a^	7 (25.0)
Neovascular	1 (3.6)
Primary open angle	6 (21.4)
Pseudoexfoliation	4 (14.3)
*Prior Surgeries, N (%)*	
None	5 (17.9)
AGI	3 (10.7)
Anterior vitrectomy	3 (10.7)
DSEK/DSAEK	4 (14.3)
KPro	3 (10.7)
iStent^b^	1 (3.6)
Phaco	20 (71.4)
PKP	2 (7.1)
PPV	8 (28.6)
Trabeculectomy	4 (14.3)
Other (OGI repair, GSL, EL)	7 (25.0)
*Prior Glaucoma Laser, N (%)*	
None	8 (28.6)
ALT	1 (3.6)
LTP	1 (3.6)
LPI	5 (17.9)
MPCPC/CWCPC	10 (35.7)
SLT	3 (10.7)
PRP	1 (3.6)
YAG	3 (10.7)
*Preoperative Baseline*	
IOP (mmHg)	
Mean ± SD	22.8 ± 7.3
Range	10.5–38
# of Glaucoma Medications	
Mean ± SD	4.3 ± 1.0
Range	2–6
Visual Acuity (LogMAR)	
Mean ± SD	0.94 ± 0.96
Range	0–3
*Surgical Indications, N (%)*	
Aphakia	6 (21.4)
ACIOL	5 (17.9)
Chronic angle-closure glaucoma	9 (32.1)
DSEK/DSAEK	4 (14.3)
KPro	3 (10.7)
Vitreous prolapse	3 (10.7)
Lens fragment in vitreous	2 (7.1)
PDR with VH	3 (10.7)
Hypertensive retinopathy with VH	1 (3.6)
*Type of Procedure, N (%)*	
AGI / PPV	24 (85.7)
BGI / PPV	4 (14.3)
*PPV Gauge, N (%)*	
23G	9 (32.1)
25G	19 (67.9)

*N* number of eyes, *SD* standard deviation, *IOP* intraocular pressure, *mmHg* millimeters of mercury, *LogMAR* logarithm of the minimum angle of resolution, *AGI* Ahmed glaucoma implant, *BGI* Baerveldt glaucoma implant, *PPV* pars plana vitrectomy, *ALT* argon laser trabeculoplasty, *LTP* laser trabeculoplasty, *LPI* laser peripheral iridotomy, *MPCPC* micropulse cyclophotocoagulation, *CWCPC* continuous wave cyclophotocoagulation, *SLT* selective laser trabeculoplasty, *PRP* panretinal photocoagulation, *YAG* YAG capsulotomy, *DSEK* Descemet stripping endothelial keratoplasty, *DSAEK* Descemet stripping automated endothelial keratoplasty, *KPro* keratoprosthesis, *Phaco* phacoemulsification, *PKP* penetrating keratoplasty, *OGI* open globe injury, *GSL* goniosynechialysis, *EL* endolaser, *ACIOL* anterior chamber intraocular lens, *PDR* proliferative diabetic retinopathy, *VH* vitreous hemorrhage

^a^Mixed mechanism glaucoma includes a combination of primary open angle, chronic angle closure, steroid response, pseudoexfoliative, traumatic, uveitic, and neovascular glaucoma as well as glaucoma secondary to an iris melanoma or corneal transplantation

^b^iStent Trabecular Micro-Bypass Stent (Models GTS100R and GTS100L, Glaukos Corporation, San Clemente, California); and iStent *inject®* Trabecular Micro-Bypass System (Model G2-M-IS, Glaukos Corporation, San Clemente, California)

The type of implant and PPV gauge are listed in Table 1. Twenty-four eyes (85.7%) received an Ahmed glaucoma implant, and the remaining 4 eyes (14.3%) received a Baerveldt glaucoma implant. A 25G vitrectomy was performed in 19 eyes (67.9%) and 23G vitrectomy in 9 eyes (32.1%).

**Fig. 1:**
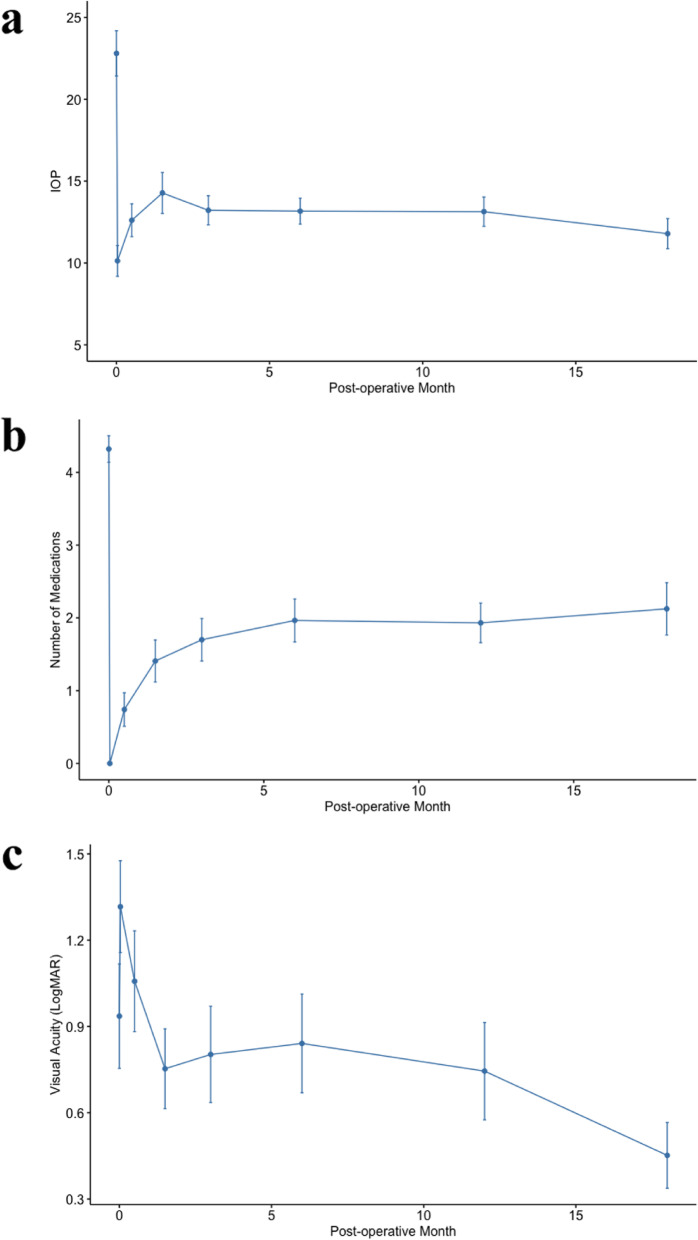
Line graph of average values of postoperative (**a**) intraocular pressure, (**b**) number of medications, and (**c**) visual acuity over time for glaucoma patients who underwent pars plana glaucoma drainage device implantation with pars plana vitrectomy using the vitrectomy sclerotomy for tube insertion. Error bars denote standard deviation of the mean

Postoperative intraocular pressure, medication burden, and visual acuity outcomes are summarized in Table 2. Line graphs of postoperative outcomes are shown in Fig. 1. IOP was significantly decreased at all follow-up time points compared to preoperative levels, with an average reduction of 7.7 ± 5.6 mmHg at POY1.5 (*p* = 0.002). Medication burden was also significantly decreased at all time points with an average of 2.1 ± 1.2 at POY1.5 (*p* = 0.004). VA was unchanged from preoperative levels at all time points after POD1. VA was the same or improved at the last follow-up visit for 23 eyes (82.1%), with the remaining 5 eyes (17.9%) experiencing decreased vision. One eye experienced VA loss by 1 line, 2 eyes by 2 lines, and 2 eyes by > 2 lines. One eye that experienced VA loss of > 2 lines had previously undergone placement of a Boston keratoprosthesis type 1 with a postoperative course complicated by endophthalmitis, chronic angle-closure glaucoma, and a persistent corneal epithelial defect. The other eye with VA loss of > 2 lines underwent Descemet stripping epithelial keratoplasty after the tube insertion surgery due to persistent corneal edema from aphakic bullous keratopathy, which was present preoperatively.

**Table 2:** Intraocular pressure, medication burden, and visual acuity outcomes data at different time points for glaucoma patients who underwent pars plana glaucoma drainage device implantation with pars plana vitrectomy using the vitrectomy sclerotomy for tube insertion

	IOP (mmHg)	Medications	VA (LogMAR)^a^
*Preoperative (n = 28)*			
Mean (SD)	22.8 (7.3)	4.3 (1.0)	0.94 (0.96)
*1 day (n = 27)*			
Mean (SD)	10.1 (4.9)	0.0 (0.0)	1.32 (0.80)
Decrease from baseline	12.2 (8.9)	4.3 (1.0)	−0.39 (0.86)
*p* value	< 0.001*	< 0.001*	0.031*
*2 weeks (n = 27)*			
Mean (SD)	12.6 (5.2)	0.7 (1.2)	1.06 (0.89)
Decrease from baseline	10.3 (9.7)	3.6 (1.4)	−0.20 (0.96)
*p* value	< 0.001*	< 0.001*	0.280
*6 weeks (n = 27)*			
Mean (SD)	14.3 (6.5)	1.4 (1.5)	0.75 (0.69)
Decrease from baseline	8.9 (7.8)	2.9 (1.5)	0.21 (0.75)
*p* value	< 0.001*	< 0.001*	0.211
*3 months (n = 25)*			
Mean (SD)	13.2 (4.4)	1.7 (1.5)	0.80 (0.82)
Decrease from baseline	10.4 (7.9)	2.6 (1.3)	0.17 (0.84)
*p* value	< 0.001*	< 0.001*	0.486
*6 months (n = 24)*			
Mean (SD)	13.2 (3.9)	2.0 (1.4)	0.84 (0.82)
Decrease from baseline	10.2 (7.6)	2.2 (1.4)	0.20 (0.98)
*p* value	< 0.001*	< 0.001*	0.433
*1 year (n = 22)*			
Mean (SD)	13.1 (4.2)	1.9 (1.3)	0.74 (0.78)
Decrease from baseline	9.4 (8.3)	2.2 (1.4)	0.12 (1.03)
*p* value	< 0.001*	< 0.001*	0.570
*1.5 years (n = 12)*			
Mean (SD)	11.8 (3.2)	2.1 (1.2)	0.45 (0.38)
Decrease from baseline	7.7 (5.6)	2.1 (1.2)	0.57 (1.03)
*p* value	0.002*	0.004*	0.185

*IOP* intraocular pressure, *mmHg* millimeters of mercury, *VA* visual acuity, *LogMAR* logarithm of the Minimum Angle of Resolution, *n* number of eyes, *SD* standard deviation

^a^Patients with LP or NLP at these time points were excluded in mean and *p* value calculations due to a lack of a validated LogMAR equivalent.

*Indicates significant *p* value < 0.05

**Fig. 2:**
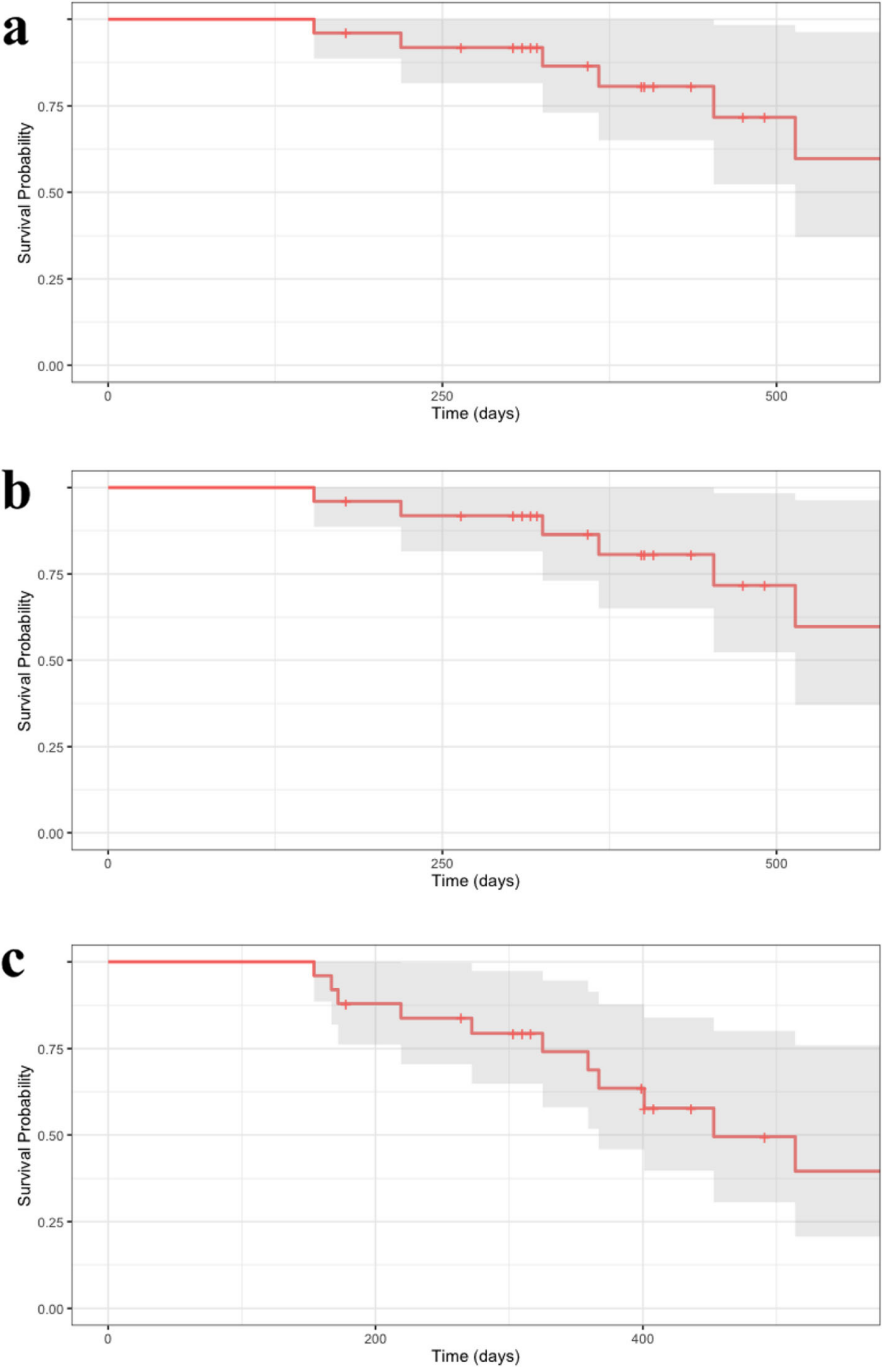
Kaplan-Meier survival curves of pars plana glaucoma drainage device insertion with pars plana vitrectomy using one of the vitrectomy port sites for tube placement. Success criteria were defined as the following: postoperative intraocular pressure (IOP) reduction ≥ 20% with IOP > 5 mmHg and (**a**) IOP ≤ 21 mmHg; or (**b**) IOP ≤ 18 mmHg; or (**c**) IOP ≤ 14 mmHg without additional IOP-lowering glaucoma procedures or loss of light perception vision. A failure was recorded on the latter visit if a patient failed to meet success criteria on two consecutive follow-up visits after 3 months

Cumulative success probabilities derived from Kaplan-Meier analyses for all three success criteria are shown in Table 3, with corresponding Kaplan-Meier curves depicted in Fig. 2. For success defined as IOP ≤ 21 mmHg or ≤ 18 mmHg (Criteria 1 and 2, respectively) as above, success probabilities were both 86.4 ± 7.4% at 1 year and 59.8 ± 14.6% at 1.5 years. For an even stricter success criteria of IOP ≤ 14 mmHg (Criteria 3), success probability was 68.8 ± 10.0% at 1 year and 39.6 ± 13.2% at 1.5 years. Hazard ratios for age, sex, glaucoma stage, family history of glaucoma, type of glaucoma implant, vitrectomy cannula gauge, preoperative IOP, or preoperative medication burden were not significant for any success criteria and are listed in Table 4.

**Table 3:** Cumulative success probabilities at different time points based on Kaplan-Meier survival analyses

	Cumulative Success (%) ± SE (95% Confidence Interval)		
	IOP reduction ≥ 20% with IOP > 5 mmHg and		
	IOP ≤ 21 mmHg	IOP ≤ 18 mmHg	IOP ≤ 14 mmHg
**3 months**	100.0 ± 0.0	100.0 ± 0.0	100.0 ± 0.0
(n = 25)	(100.0, 100.0)	(100.0, 100.0)	(100.0, 100.0)
**6 months**	96.0 ± 3.9	96.0 ± 3.9	88.0 ± 6.5
(n = 24)	(88.6, 100.0)	(88.6, 100.0)	(76.1, 100.0)
**1 year**	86.4 ± 7.4	86.4 ± 7.4	68.8 ± 10.0
(*n* = 17)	(73.1, 100.0)	(73.1, 100.0)	(51.8, 91.4)
**1.5 years**	59.8 ± 14.6	59.8 ± 14.6	39.6 ± 13.2
(*n* = 8)	(37.1, 96.3)	(37.1, 96.3)	(20.7, 75.9)

*SE* standard error, *IOP* intraocular pressure; *n* number of eyes

**Table 4:** Hazard ratios from univariate Cox proportional-hazard modeling using demographic and preoperative data

**Parameter**	**IOP reduction ≥ 20% with 5 < IOP ≤ 21 mmHg**	
	**HR (95% CI)**	***p*** **value**
Age	0.979 (0.931–1.031)	0.4
Sex (Female)		0.6
Male	0.619 (0.113–3.407)	
Glaucoma stage (Indeterminate)		0.3
Mild	0.189 (0.011–3.367)	
Moderate	0.331 (0.030–3.616)	
Severe	0.066 (0.004–1.137)	
Family history of glaucoma (No)		1.0
Yes	0.972 (0.097–9.784)	
Type of glaucoma implant (AGI)		0.9
BGI	0.834 (0.094–7.364)	
Vitrectomy cannula gauge	0.973 (0.175–5.408)	1.0
Preoperative IOP	0.864 (0.722–1.033)	0.1
Preoperative medication burden	0.465 (0.184–1.175)	0.1
**Parameter**	**IOP reduction ≥ 20% with 5 < IOP ≤ 18 mmHg**	
	**HR (95% CI)**	***p*** **value**
Age	0.979 (0.931–1.031)	0.4
Sex (Female)		0.6
Male	0.619 (0.113–3.407)	
Glaucoma stage (Indeterminate)		0.3
Mild	0.189 (0.011–3.367)	
Moderate	0.331 (0.030–3.616)	
Severe	0.066 (0.004–1.137)	
Family history of glaucoma (No)		1.0
Yes	0.972 (0.097–9.784)	
Type of glaucoma implant (AGI)		0.9
BGI	0.834 (0.094–7.364)	
Vitrectomy cannula gauge	0.973 (0.175–5.408)	1.0
Preoperative IOP	0.864 (0.722–1.033)	0.1
Preoperative medication burden	0.465 (0.184–1.175)	0.1
**Parameter**	**IOP reduction ≥ 20% with 5 < IOP ≤ 14 mmHg**	
	**HR (95% CI)**	***p*** **value**
Age	0.987 (0.952–1.023)	0.5
Sex (Female)		0.1
Male	2.697 (0.771–9.438)	
Glaucoma stage (Indeterminate)		0.4
Mild	0.410 (0.035–4.753)	
Moderate	0.751 (0.084–6.721)	
Severe	0.228 (0.023–2.289)	
Family history of glaucoma (No)		0.6
Yes	0.431 (0.082–2.263)	
Type of glaucoma implant (AGI)		0.4
BGI	0.397 (0.050–3.175)	
Vitrectomy cannula gauge	1.114 (0.292–4.256)	0.9
Preoperative IOP	1.021 (0.934–1.116)	0.6
Preoperative medication burden	0.874 (0.436–1.753)	0.7

*IOP* intraocular pressure, *HR* hazard ratio, *CI* confidence interval, *AGI* Ahmed glaucoma implant, *BGI* Baerveldt glaucoma implant

Last follow-up visit outcomes are listed in Table 5. Three eyes (10.7%) achieved complete success and 14 eyes (50.0%) achieved qualified success under Criteria 1. Four eyes (14.3%) were a qualified failure and 7 eyes (25.0%) required additional glaucoma surgery, as detailed below.

**Table 5:** Summary of intraocular pressure, medication burden, and visual acuity outcomes at the last follow-up visit for glaucoma patients who underwent pars plana glaucoma drainage device implantation with pars plana vitrectomy using the vitrectomy sclerotomy for tube insertion

	IOP (mmHg)	Medications	VA (LogMAR)
*Total*^a^ *(n = 21)*			
Mean ± SD	12.0 ± 3.7	2.3 ± 1.4	0.79 ± 0.75
Range	6–20	0–4	0–3
*Complete success (n = 3)*			
Mean ± SD	8.7 ± 1.5	0 ± 0	0.51 ± 0.05
Range	7–10	0	0.48–0.54
*Qualified success (n = 14)*			
Mean ± SD	12.0 ± 3.9	2.4 ± 1.0	0.80 ± 0.80
Range	6–20	1–4	0.10–3
*Qualified failure (n = 4)*			
Mean ± SD	14.8 ± 1.3	3.5 ± 0.6	0.89 ± 0.86
Range	13–16	3–4	0–2

*IOP* intraocular pressure, *mmHg* millimeters of mercury, *VA* visual acuity, *LogMAR* logarithm of the Minimum Angle of Resolution, *n* number of eyes, *SD* standard deviation

^a^Excluding eyes that underwent additional procedures after the initial combined procedure and were categorized as failures

Complication rates are shown in Table 6. The most common complications prior to 3 months postoperatively were anterior chamber (AC) inflammation in 13 eyes (46.4%), corneal edema in 9 eyes (32.1%), and cystoid macular edema (CME) in 5 eyes (17.9%). Late complications after 3 months included CME in 5 eyes (20.8%), corneal edema in 3 eyes (12.5%), and AC inflammation in 1 eye (4.2%). Seven of the 9 eyes with early corneal edema developed corneal abrasions intraoperatively that resolved prior to 3 months postoperatively, and the remaining 2 eyes had preoperative corneal edema with a history of penetrating keratoplasty. These two eyes also had prolonged corneal edema postoperatively, which persisted through 6 months postoperatively in one eye due to a combination of Fuch’s dystrophy, aphakia, and preoperative vitreous prolapse and at 8 months postoperatively in the second eye due to a persistent epithelial defect. A third eye with prolonged corneal edema had aphakic bullous keratopathy at 9 months postoperatively requiring a Descemet stripping automated endothelial keratopathy the following month. Five eyes had CME after surgery, which was present pre-operatively in three eyes and resolved in one eye at 2 months, the second eye at 3 months, and the third eye at 9 months postoperatively. In the two remaining eyes, one eye developed CME at 3 months, which resolved after 2 months of topical steroid use. In the other eye, CME developed at 1.5 months and persisted through their last follow-up despite topical steroid use.

**Table 6:** Postoperative early and late complication rates for glaucoma patients who underwent pars plana glaucoma drainage device implantation with pars plana vitrectomy using the vitrectomy sclerotomy for tube insertion

	N (%)					
	AC Inflammation	Hypotony	Corneal edema	Cystoid macular edema	Vitreous hemorrhage	Tube obstruction
**Total (*n*** **= 28)**	14 (50.0)	1 (3.6)	9 (32.1)	5 (17.9)	6 (21.4)	2 (7.1)
**Early**^a^ **(*n*** **= 28)**	13 (46.4)	1 (3.6)	9 (32.1)	5 (17.9)	4 (14.3)	2 (7.1)
**Late**^b^ **(*n*** **= 24)**	1 (4.2)	0 (0.0)	3 (12.5)	5 (20.8)	0 (0.0)	0 (0.0)

*N* number of eyes in group, *n* total number of eyes, *AC* anterior chamber

^a^Complications present up to 3 months postoperatively, not including preoperative findings

^b^Complications present after 3 months postoperatively

Hypotony was noted in 1 patient (3.6%) and self-resolved after 2 weeks. Vitreous hemorrhage was present in three eyes prior to surgery and resolved with surgery in two eyes. Vitreous hemorrhage was found in 4 eyes postoperatively and self-resolved after 2 weeks. One eye with preoperative vitreous hemorrhage and one eye with a diagnosis of NVG but no preoperative vitreous hemorrhage received an intravitreal bevacizumab injection one month prior to the combined PP GDD and PPV procedure. Two eyes (7.1%) required subsequent PPV for tube obstruction with vitreous or blood. No eyes developed choroidal detachment, retinal tears, retinal detachment, endophthalmitis, or diplopia postoperatively.

Seven patients had additional glaucoma surgeries after the combined surgery. Two patients received a combination of augmented Micropulse transscleral cyclophotocoagulation (MP-TSCPC) [11] and continuous wave transscleral cyclophotocoagulation at 15 months and 16 months respectively. Another two patients underwent MP-TSCPC at 2.5 months, and one patient underwent MP-TSCPC at 8.2 months. Finally, one patient underwent PPV at 1 month for vitreous occluding the tube, and another patient underwent PPV at 2 months for blood occluding the tube.

## Discussion

This study is the largest study to date that examines outcomes of pars plana GDD insertion through an existing vitrectomy sclerotomy site. In this study, the mean IOP and medication burden were significantly reduced at all postoperative time points compared to preoperative levels. Three eyes achieved complete success and 14 eyes achieved qualified success at their last follow-up visit, for a total success rate of 60.7% (17/28 eyes) under Criteria 1. This success rate is slightly lower than total success rates reported by prior studies ranging from 67 to 100%, as seen in Tables 7, 8, and 9. However, given that our mean preoperative IOP was lower than that of other studies, our lower success rate may instead potentially reflect a lower magnitude of IOP reduction secondary to a lower starting IOP. Vitrectomy gauge was not a predictive factor for failure, as demonstrated by the nonsignificant hazard ratio. Average visual acuity was unchanged from preoperative levels at all follow-up visits after POD1, and two patients experienced > 2 lines of visual acuity loss due to corneal problems as previously described in our results. Overall, the final postoperative IOP, postoperative medication burden, and visual acuity findings in this study were similar to prior studies evaluating outcomes of combined pars plana GDD insertion and vitrectomy **(**Tables 7, 8, and 9**)**.

**Table 7:** Selection of retrospective studies of pars plana glaucoma drainage device placement in a new sclerotomy separate from the vitrectomy sclerotomy sites

Author, year	Tarantola et al., 2011 [15]	Shaikh et al., 2014 [16]	Varma et al., 1995 [17]	Sidoti et al., 2001 [18]	Witmer et al., 2010 [19]
**Publication**	Retina	BMJ Ophthal	AJO	Ophthal	J Glaucoma
**Glaucoma type**	Uncontrolled CACG	Mixed	OAG and ACG	Mixed	Mixed
**GDD type**	Baerveldt	–	Baerveldt	Ahmed, Baerveldt, Molteno, Krupin	Baerveldt
**Sclerotomy for tube insertion**	Different	Different	Different	Different	Different
**PPV gauge**	20 (endoscope-assisted)	20, 23, 25 (endoscope-assisted)	–	–	–
**F/U (months)**	Mean 62 (range 10–106)	Median 18	(range 12–28)	Mean 12.1 (range 0–31.8)	Mean 38.4 (range 6–86)
**# of eyes**	19	13	13	34	51
**% Complete / qualified success**^a^	26 / 47	–	–	47 / 41	–
**IOP (mean preop / final (mmHg))**	31.3 / 11.4	23.0 / 12.0	35 / 13	17.9 / 15.1	26.9 / 13.5
**# of Meds (mean preop / final)**	3.4 / 1.3	3.1 / 0.3	- / 0.8	–	3.2 / 1.1
**# VA decline**	1 (5.3%)	–	4 (30.8%)	5 (15%)	16 (31.4%)
**# eyes with complications**	5 (26.3%)	0 (0%)	–	–	20 (30%)
**Select complications (%)**	vTB – 5; sTB – 5; SR – 5; hCD – 5	–	LEM – 15	CGF – 50; H – 3; CD – 12; CME – 3; RD – 6; vTB – 9; bTB – 3; VH – 6; U – 6; Hy – 3; ScH – 6	I – 10; RD – 6; D – 6; vTB – 4; H – 4; ERM – 4; CEf – 4
**# of glaucoma reoperations**	5 (26.3%)	3 (23.1%)	2 (15.4%)	–	–

*CACG* chronic angle closure glaucoma, *OAG* open angle glaucoma, *ACG* acute-closure glaucoma, *vTB* vitreal tube blockage, *sTB* swollen Soemmering’s ring blocking tube, *SR* shunt retraction, *CD* choroidal detachment, *hCD* hemorrhagic choroidal detachment, *LEM* limited eye movement, *D* diplopia, *vTB* vitreal tube blockage, *H* hypotony, as defined by IOP < 6 mmHg; *ScH* suprachoroidal hemorrhage, *Hy* hyphema, *CGF* corneal graft failure, *U* uveitis, *ERM* epiretinal membrane, *I* iritis, *CEf* choroidal effusion, *CME* cystoid macular edema, *RD* retinal detachment

^a^Complete success was defined as IOP between 6 and 21 mmHg without medications. Qualified success was defined as IOP between 6 and 21 mmHg with medications

**Table 8:** Selection of retrospective studies of pars plana glaucoma drainage device placement in either a new sclerotomy or existing vitrectomy sclerotomy site

Author, year	de Guzman et al., 2006 [20]	Qin et al., 2018 [21]	Kolomeyer et al., 2015 [6]	Kolomeyer et al., 2012 [22]	Kaynak et al., 1998 [23]	Luttrull et al., 2000 [24]
**Publication**	Clin Exp Ophthal	J Glaucoma	Retina	Oman J Ophthal	Br J Ophthal	Ophthal
**Glaucoma type**	Mixed	Mixed	NVG	Mixed	Mixed (no NVG)	Mixed
**GDD type**	Baerveldt, Molteno	Ahmed, Baerveldt	Baerveldt	Baerveldt	Molteno	Baerveldt
**Sclerotomy for tube insertion**	Both	Both	Both	Both	Unspecified	Unspecified
**PPV gauge**	–	–	20, 23, 25	20, 23	–	–
**F/U (months)**	Mean 30.2 (range 6–77)	Mean 43.5	Mean 19.9 (range 2–66)	Mean 33.7	Mean 30.3 (range 4–71)	Mean 18 (range 3–41)
**# of eyes**	33	57	89	39	17	50
**% Complete / qualified success**^a^	49 / 42	–	30 / 37	21 / 54	–	56 / 28
**IOP (mean preop / final (mmHg))**	33.1 / 13.4	29.0 / 15.1	37.2 / 15.9	31.9 / 13.2	–	44 / 14
**# of Meds (mean preop / final)**	3.6 / 0.6	2.9 / 1.1	2.8 / 1.21	3.8 / 1.7	–	3.2 / 0.6
**# VA decline**	13 (39.4%)	22 (38.6%)	34 (38%)	14 (36%)	2 (11.8%)	14 (28%)
**# eyes with complications**	–	16 (28.1%)			8 (47.1%)	
**Select complications (%)**	VH/H – 12; CD – 27; CE – 30; U – 3; LEM – 3; CGF – 15; vTB – 6; iTB – 3; ERM – 3; CEf – 3	D – 16; H – 9; VH – 2; TE – 2	OH – 92; H – 22; Hy – 21; CE – 19; VH – 16; CME – 15; CD – 12; RD – 4; TB – 3; En – 3; SrH – 1; ERM – 2	H – 23; CD – 26; OH – 13; RH – 10; VH – 5; CME – 5; ERM – 5	H – 12; VH – 6; CD – 6; RD – 12; CED – 29	CEf – 36; CME – 4; D – 6; RD – 8; CH – 4; VH – 2
**# of glaucoma reoperations**	13 (39.4%)	8 (14.0%)	9 (10%)	5 (13%)	1 (5.9)	–

*NVG* neovascular glaucoma, *VH* vitreal hemorrhage, *CD* choroidal detachment, *CE* corneal edema, *U* uveitis, *LEM* limited eye movement, *CGF* corneal graft failure, *vTB* vitreal tube blockage, *iTB* iris blocking tube, *ERM* epiretinal membrane, *CEf* choroidal effusion, *D* diplopia, *H* hypotony, as defined by IOP < 6 mmHg; *TE* tube erosion, *OH* ocular hypertension, *Hy* hyphema, *RD* retinal detachment, *TB* tube blockage, *En* endophthalmitis, *SrH* subretinal hemorrhage, *RH* retinal hemorrhage, *CED* corneal endothelial decompensation, *CEf* choroidal effusion, *CH* choroidal hemorrhage

^a^Complete success was defined as IOP between 6 and 21 mmHg without medications. Qualified success was defined as IOP between 6 and 21 mmHg with medications

**Table 9:** Selection of retrospective studies of pars plana glaucoma drainage device placement in an existing vitrectomy sclerotomy site

Author, year	Reichstein et al., 2011 [7]	Kolomeyer et al., 2012 [5]	Present study
**Publication**	Ophthal	Eur J Ophthal	–
**Glaucoma type**	Mixed	Mixed	Mixed
**GDD type**	Ahmed, Baerveldt	Baerveldt	Ahmed, Baerveldt
**Sclerotomy for tube insertion**	Same	Same	Same
**PPV gauge**	25	23	23, 25
**F/U (months)**	> 12	Mean 12.1 (range 6–27)	Mean 14.2 (range 3.1–35.5)
**# of eyes**	10	8	28
**% Complete / qualified success**^a^	–	25 / 75	14 / 46
**IOP (mean preop / final (mmHg))**	31 / 16.1	29.1 / 13.8	22.8 / 11.8
**# of Meds (mean preop / final)**	2.5 / -	3.9 / 1.9	4.3 / 2.0
**# VA decline**	3 (30%)	3 (37.5%)	7 (25%)
**# eyes with complications**	–	–	14 (50%)
**Select complications (%)**	CE – 20	H – 38; DR – 25; VH – 13; IRH – 25; CE – 13; CH – 13; CD – 25	ACI – 50; H – 4; CE – 32; CME – 18; VH – 21; vTB – 4; bTB – 4
**# of glaucoma reoperations**	0 (0.0%)	1 (12.5%)	6 (21.4%)

*CE* corneal edema, *H* hypotony, as defined by IOP < 6 mmHg; *DR* decompression retinopathy, *VH* vitreal hemorrhage, *IRH* intraretinal hemorrhage, *CH* choroidal hemorrhage, *CD* choroidal detachment, *ACI* anterior chamber inflammation, *vTB* vitreal tube blockage, *bTB* blood tube blockage

^a^Complete success was defined as IOP between 6 and 21 mmHg without medications. Qualified success was defined as IOP between 6 and 21 mmHg with medications

The most common complication encountered in this study was transient anterior chamber inflammation in 13 out of 28 eyes (46.4%), which resolved in all cases after 2 weeks except for one eye, where inflammation was present intermittently and resolved after 1 year. Corneal edema was present in 2 eyes preoperatively and developed in 7 eyes postoperatively. Edema was present in 3 eyes by 6 weeks and resolved in all eyes after 1 year. Corneal edema was most commonly due to corneal abrasions acquired during surgery, although a component of prolonged surgical insult from the combined PPV and GDD surgeries may also have contributed. Prolonged corneal edema has also been shown to result from PPV alone [12, 13]. These complication rates were comparable to that of prior studies, as seen in Tables 7, 8, and 9.

Previously, GDD tube insertion in an existing vitrectomy sclerotomy was thought to result in a higher risk of hypotony due to aqueous leakage around the tube, particularly when sclerotomies larger than 21-gauge were used for vitrectomy [3, 6]. Scott et al. [3] demonstrated a higher incidence of hypotony in their eyes with GDD tubes inserted into sclerotomies created by 20-gauge needles (0/8 eyes) compared to 23-gauge needles (5/18 eyes, 28%). In a study of neovascular glaucoma patients, Kolomeyer et al. [6] also noted a significantly higher rate of transient hypotony in 20-gauge versus 23- or 25-gauge PPV eyes (*p* = 0.021), with tube insertion into an existing sclerotomy for the 23- and 25-gauge PPV. In comparison, for 25-gauge PPV, Reichstein et al. [7] found no hypotony amongst the 10 eyes studied also with GDD tube placement in a vitrectomy sclerotomy. In our study, hypotony was noted in 1 eye (3.6%) and self-resolved after 2 weeks, similar to the low rates of hypotony noted in prior studies with small-gauge PPV regardless of sclerotomy status. Thus, tube insertion in an existing sclerotomy site from 23- or 25-gauge PPV may result in a lower risk of hypotony compared with 20-gauge PPV, and creating an additional sclerotomy for the GDD tube may not be necessary.

Vitreous hemorrhage was found in a total of 6 eyes (21.4%) and was present in 3 eyes prior to surgery. Three additional eyes developed vitreous hemorrhage postoperatively, which resolved after 2 weeks. Of the eyes that had vitreous hemorrhage preoperatively, two resolved with surgery, and one resolved after 2 weeks. Two eyes (7.1%) experienced tube occlusion with either blood or vitreous requiring subsequent PPV. These findings of a 12% (3/25 eyes) incidence of vitreous hemorrhage are within the range of findings from prior studies of PP GDD placement and PPV **(**Tables 7, 8, and 9**)**. A higher incidence of vitreous hemorrhage has also been noted in studies of patients with neovascular glaucoma, with Kolomeyer et al. [6] reporting 14 out of 89 eyes (16%) and Campagnoli et al. [14] reporting 25 out of 43 eyes (58.1%) with vitreous hemorrhage on postoperative day 1 persisting through 1 year in 16 eyes.

The postoperative prevalence of CME was slightly higher in our study than in prior studies. Five eyes (17.9%) had CME postoperatively with resolution in 3 eyes prior to 1 year. However, given that 3 eyes had pre-existing CME prior to surgery, the true incidence of CME within our study is 2 out of the 23 at-risk eyes in our cohort (8.7%), congruent with results from prior studies of pars plana GDD insertion and vitrectomy demonstrating rates between 3 to 15% **(**Tables 7, 8, and 9**)**. Given that Massachusetts Eye and Ear is a tertiary-care center and that 3 of the 5 eyes with postoperative CME had CME preoperatively, it is possible that the patients in our study were sicker and more complex than patients at non-tertiary-care centers, potentially resulting in our higher prevalence of CME compared to other studies. Other retinal complications from pars plana vitrectomy, including retinal detachment, retinal tears, and retinal dialyses, were not observed in our study.

Limitations of this study include its retrospective design, small sample size, lack of a comparison group of tube insertions in a separate sclerotomy, large number of surgeons, and short follow-up time period for patients who had undergone surgery more recently, particularly as expected follow-up visits were displaced by COVID-19. This study is likely not generalizable to surgeries that were not Ahmed valves or if the implant were placed in a location other than the superotemporal quadrant. As children were not included in our study, this study is not applicable to that population. Smaller sample sizes at later time points may have affected the significance of statistical testing. Given that multiple glaucoma and retina surgeons performed the procedure, it is less likely that bias from a single surgeon affected the results of our study; however, the variability in surgical technique may affect our results. Despite extensive efforts to identify a comparison group with tube insertions in a new sclerotomy separate from the vitrectomy sclerotomy sites, we could not identify a sufficient number of cases to form a suitable comparison group for this study. Thus, we relied on comparisons with prior studies of pars plana GDD insertion and vitrectomy to evaluate our results. Finally, there may be a referral bias as patients in our study were treated at a tertiary-care center, which may limit the generalizability of our results.

## Conclusions

In summary, our results demonstrate that inserting a GDD tube into an existing vitrectomy sclerotomy site during combined pars plana GDD insertion and small-gauge vitrectomy likely does not increase the risk of complications. Specifically, there does not appear to be an increased risk of hypotony through leakage around the tube when an existing vitrectomy sclerotomy is utilized. We found similar functional outcomes and complication risk compared to prior studies of the same combined surgery, regardless of tube placement in existing or new sclerotomies. Minimizing the number of sclerotomies created during surgery may potentially reduce the risk of hypotony or tissue trauma and decrease operative time. Thus, GDD placement in a vitrectomy sclerotomy can be safely considered in cases that utilize small-gauge pars plana vitrectomy.

## Data Availability

The data that support the findings of this study are available on request from the corresponding author DS. The data are not publicly available due to them containing information that could compromise research participant privacy.

## Abbreviations

PP: Pars plana
GDD: Glaucoma drainage device
PPV: Pars plana vitrectomy
IOP: Intraocular pressure
BCVA: Best-corrected visual acuity
VA: Visual acuity
POD1: Postoperative day 1
POW2: Postoperative week 2
POW6: Postoperative week 6
POM3: Postoperative month 3
POM6: Postoperative month 6
POY1: Postoperative year 1
POY1.5: Postoperative year 1.5
CME: Cystoid macular edema
NLP: No light perception
SD: Standard deviation
LogMAR: Logarithm of the minimum angle of resolution
MP-TSCPC: Micropulse transscleral cyclophotocoagulation
